# 3-Dimensional Diffusion Tensor Imaging (DTI) Atlas of the Rat Brain

**DOI:** 10.1371/journal.pone.0067334

**Published:** 2013-07-05

**Authors:** Ashley Rumple, Matthew McMurray, Josephine Johns, Jean Lauder, Pooja Makam, Marlana Radcliffe, Ipek Oguz

**Affiliations:** 1 UNC Chapel Hill, Chapel Hill, North Carolina, United States of America; 2 UIC, Chicago, Illinois, United States of America; 3 UI, Iowa City, Iowa, United States of America; University of Medicine & Dentistry of NJ - New Jersey Medical School, United States of America

## Abstract

Anatomical atlases play an important role in the analysis of neuroimaging data in rodent neuroimaging studies. Having a high resolution, detailed atlas not only can expand understanding of rodent brain anatomy, but also enables automatic segmentation of new images, thus greatly increasing the efficiency of future analysis when applied to new data. These atlases can be used to analyze new scans of individual cases using a variety of automated segmentation methods. This project seeks to develop a set of detailed 3D anatomical atlases of the brain at postnatal day 5 (P5), 14 (P14), and adults (P72) in Sprague-Dawley rats. Our methods consisted of first creating a template image based on fixed scans of control rats, then manually segmenting various individual brain regions on the template. Using itk-SNAP software, subcortical and cortical regions, including both white matter and gray matter structures, were manually segmented in the axial, sagittal, and coronal planes. The P5, P14, and P72 atlases had 39, 45, and 29 regions segmented, respectively. These atlases have been made available to the broader research community.

## Introduction

In recent years, small animal neuroimaging studies, especially magnetic resonance imaging (MRI) and diffusion tensor imaging (DTI) have become increasingly popular. Unlike many clinical studies, animal models allow for tight experimental control over genetics, nutrition, and subject compliance to treatment, making it possible for researchers to carry out more focused experiments with fewer variables. MRI and DTI provide particularly useful tools for this purpose, since both the data acquisition and analysis techniques are highly translational.

DTI is a relatively recent MR imaging technique [1] that allows for the measurement of microstructural characteristics of tissue. By applying a series of magnetic field gradients and fitting a mathematical model to the multidimensional data thus acquired, DTI provides a representation of the local water diffusion properties in the tissue in a directionally-dependent manner. This is particularly useful in the brain, where white matter typically has a highly preferential diffusion direction (i.e. more diffusion along the axons rather than perpendicular to them). DTI allows quantifying this level of directional dependency or anisotropy via metrics such as the fractional anisotropy (FA). Additional measures are the parallel and perpendicular components of the amount of diffusion, namely, the axial diffusivity (AD) and the radial diffusivity (RD), as well as the bulk amount of diffusion regardless of the diffusion direction, i.e. the mean diffusivity (MD). Finally, DTI also allows for measurement of the principal direction of diffusion; this data can be leveraged for tractography and connectivity studies that investigate the connections in the brain on a more global scale [2].

DTI is of particular interest in neurodevelopmental studies; as it can produce better contrast than structural MRI between white matter and gray matter for un-myelinated tissue in the developing brains of young pups. Thus, unlike MRI, DTI can produce distinct contrast between tissue types even in embryonic and young neonatal ages [3]. In the human brain imaging context, DTI scans often lack the level of detail necessary for accurately delineating the structures. Typical structural MR images in humans have a resolution of (1 mm)^3^, whereas typical diffusion imagery have resolutions of (2 mm)^3^ or less. However, in the postmortem animal imaging context, where ample scan time is available and motion artifacts are not a cause of concern, DTI scans can be acquired at sufficiently high resolution. The resolution used in our datasets are quite comparable to structural MRI resolution levels, making it possible to accurately segment anatomical structures. In the neurodevelopmental stages (such as our P5 and P14 atlases), using diffusion MRI rather than structural MRI has the additional advantage of showing improved contrast between white matter and gray matter structures, because this method has been shown to produce distinct contrast between tissues types even early in the maturation process where the white matter is largely unmyelinated [3]. Additionally, while anatomical images (T1/T2) can provide a single contrast with which to delineate ROIs, DTI provides a number of contrasts in the same space (FA, MD, IDWI), which can be used effectively in combination to improve the accuracy of segmentation.

There are many methods and approaches to the analysis of MRI and DTI data in rodent brain studies, a detailed review of these can be found in Oguz et al. 2012 [4]. One of the main modes of analysis is the regional analysis and comparison of tissue properties. For this type of analysis, each scan is segmented into a number of anatomical regions of interest (ROI’s), such as the hippocampus or amygdala. Given the segmentation, the volume and shape of each structure can be analyzed and compared between control and experimental groups. Additionally, in DTI datasets, diffusion properties, such as the FA, MD, AD and RD, can be measured within each ROI and compared across subjects.

The creation of such segmentations is far from trivial. Manual segmentations are subjective, tedious, extremely time-consuming, and even experienced manual raters can take days to weeks to complete the segmentation of a single scan, based on the number of ROI’s and the resolution of the scan. Recent advances in the imaging technology are allowing increasingly high throughput MR histology for the rodent brain, allowing the acquisition of very high resolution MRI datasets in a matter of hours [5]. Clearly, the bottleneck is an automated method to analyze these images. Automated segmentation methods, therefore, are crucial to the advancement of the field.

Many automated segmentation methods rely on high-resolution atlases. By using a detailed atlas previously created by an expert and registration or classification algorithms, they estimate the segmentation of new scans, in a fraction of the time it would take a manual rater. Additionally, these methods clearly have the advantage of being reproducible as well as not subjective. It should be noted that MRI- or DTI- based atlases are considerably more valuable than traditional histological atlases (such as Paxinos 2007) for the analysis of MRI/DTI data. Not only do these atlases avoid the issues of distortion that arise from the extraction of the brain from the skull and the slicing, but also they capture the full 3D nature of the MRI/DTI data and allow for addressing questions of volume, 3D shape and fiber tractography.

A crucial requirement for these automated segmentation methods is an appropriate atlas. For best results, the atlas needs to be as closely matched to the population of the study as possible. Particularly, for accurate analysis of rat brain images, it is not appropriate to use mouse atlases. While they may be relatively similar in size and anatomy, the best results for rat brain segmentations will come from using rat atlases. Also, during the early postnatal development stages, the brain undergoes a dramatic amount of change in shape and appearance in a small window of time, and using adult atlases is inadequate. Without suitable atlases, many if not all of the automated processing algorithms become inappropriate. Additionally, it is important that the atlases are constructed from a population rather than a single individual to prevent bias in the studies that use the atlas. These population atlases are commonly called variational atlases as they contain information about not just a single brain or the average brain, but also the normal variation around this average. In light of these potential confounds, the current manuscript presents three novel DTI atlases for rat brains, for two developmental ages (P5 and P14) and for adults (P72). These ages were chosen to represent considerably different time points in the developmental trajectory of the rat, roughly corresponding to the third trimester, infancy and adulthood in the human life span.

The rodent imaging community has already developed several 3D MRI mouse atlases, such as the C57 Brookhaven atlas [6], the Mouse BIRN atlas [7], the Waxholm atlas [8], the Developmental Mouse atlas [9], [10], [11], and Kovacevic et al.’s [12] atlas. The Mouse BIRN, Brookhaven, Waxholm, and the Developmental Mouse atlases are publicly available; the others might be available upon request from the authors. Recently, several rat MRI atlases with DTI acquisitions have been published as well. These include a Wistar rat atlas with postnatal time points at P0, P2, P4, P8, P12, P18, P24, P40, and P80 [13], a Sprague-Dawley rat atlas imaged at a time range of P70–P77 [14], and a Sprague-Dawley rat atlas at time point P545 [15]. Our Sprague-Dawley atlas complements these nicely, as it contributes missing time points in the rapidly changing developmental trajectory in so that, in addition to the atlases created at P70–P77 and P545, there is a wide range of ages represented by Sprague-Dawley DTI atlases available to the community.

## Materials and Methods

### 1. Ethics Statement

This study was carried out in strict accordance with the recommendations in the Guide for the Care and Use of Laboratory Animals of the National Institutes of Health. The protocol was approved by the Committee on the Ethics of Animal Experiments of the University of North Carolina (Permit Number: 27-2956). All surgery was performed under sodium pentobarbital anesthesia, and all efforts were made to minimize suffering.

### 2. Subjects

Sprague-Dawley rats served as the subjects for the creation of this atlas. All of the rats came from Charles River, Raleigh, NC, and were bred in house and cared for according to university policy and federal standards.

The P72 atlas was built from six female, nulliparous Sprague–Dawley rats (Charles River, Raleigh, NC). The average body weight for these subjects was (289.3±20.5 g). Ten additional virgin females were placed with males on a breeding rack until a sperm plug was found, which was designated as gestation day (GD) zero. Rats were then singly housed and maintained on a 12 h:12 h reverse light cycle (lights off at 0900 h) for seven days. They were then transferred to a room with a regular light cycle (lights on at 0700 h) for the remainder of the experiment, a procedure that results in the majority of dams delivering their litters during daylight hours [16]. Following delivery, litters were culled to 8 pups. One male and one female offspring from each of these 10 females were randomly selected at P5 (10.3±3.1 g) and an additional male and female selected on P14 (27.1±2.9 g) to provide the subjects for the respective atlases.

On their designated day, subjects were euthanized via a lethal dose of sodium pentobarbital and immediately underwent cardiac puncture perfusion, first with chilled 0.9% phosphate buffered saline, followed by 4% formalin. Following fixation, the whole head was removed and skin and surrounding tissue excised, leaving only the brain contained in the skull for imaging. The brain was left in the skull to avoid damage to the brain during extraction and to avoid the physical distortion that occurs when the brain is extracted. Following fixation, the head was stored in 30% sucrose at 4°C until imaging.

### 3. MRI Acquisition

An acquisition protocol was developed specifically for this study, to accommodate the unique needs of the pup imaging at these very young developmental ages. The details of this protocol are published in Cai et al. 2011 [17]. Briefly, the approach used is similar to that used by Mori et al. 1998 [18]. A 3D DTI RARE sequence with twin navigator echoes was implemented on a Bruker horizontal 9.4 T scanner (BioSpec 9.4/30 USR, Bruker Biospin, Billerica, MA, USA). The acquisition parameters were TR = 700 ms; effective TE = 23.662 ms; RARE echo spacing = 11.9 ms with a RARE factor of 3. The resolution of the P72 scans was 0.16×0.125×0.16 mm^3^; they had twelve gradient directions and two baseline images. The resolution of the P14 and P5 scans was 0.12×0.07×0.12 mm^3^ with twenty-one gradient directions and three baseline images. To account for the reduced water mobility in the tissue due to fixation, a higher b value (b = 1600s/mm^2^) was used. The total image acquisition time was 10 h.

### 4. Creation of a Template Image

Each template image was an average of the DTI scans of each age-group’s subjects. Before being averaged into a single template, each case’s image was resampled to isotropic resolution (0.07×0.07×0.07 mm^3^ for P5 and P14 and 0.125×0.125×0.125 mm^3^ for P72) and then manually skull-stripped using the software package itk-SNAP [19] so that all non-brain material was removed from the image. The MD image was used for both the skull-stripping and the subsequent registration steps; the FA and the baseline (B0) images were used for confirming the skull-strip masks in areas where the MD image appeared ambiguous.

After skull- stripping, the MD images were affinely registered and histogram-matched to a template so that the deformable registration process could be completed [20] [21]. First, the first subject’s image is used as the template, to compute an intitial average image. To minimize bias based on the ordering of the data, a second average is computed by registering affinely all the images again to the first average just obtained.

Then, the unbiased atlas was built with an iterative continuous joint optimization using fluid-based registration from the individual MD images [22]. Slicer3 was used for rigid registration; the fluid registration was performed via the AtlasWerks package (http://www.sci.utah.edu/software/13/370-atlaswerks.html). The result was a mean anatomical image that could adequately represent the anatomy of the population seen in the images. This method allows us to create an atlas that is a better representation of the anatomy of the whole population; it is inherently less biased because all the images used for making the atlas are included in the one template [22].

### 5. Segmentation of the Atlas

Once the template images were created, the atlas segmentations were created in a step-wise fashion. Initially, the structures that had the best contrast with the surrounding tissue, such as the hippocampus, the external capsule, the anterior commissure (ac), and the ventricles were segmented. Broader regions that lay on the edges of the brain such as the olfactory bulb, the cerebellum, and the brainstem were segmented next. Then, subcortical structures that did not have as strong of a contrast, but which were located next to or near structures that could be used for boundaries and landmarks were labeled. Finally, the subcortical regions that had poor contrast in the MD image were segmented.

Most of the segmentation was done in the coronal view in MD space, also using the axial and sagittal views to verify that the structures were correct with regard to the other regions in 3D space. For clarification of ambiguous areas, the FA and the IDWI images were used. We consulted the Paxinos and Watson 2007 [23] histological atlas for reference. In the segmentation of a single atlas, such as our case, it is crucial to avoid bias from having a single rater. For this purpose, rather than having the atlas segmented multiple times, a team of 6 anatomical experts confirmed each and every region’s segmentation. Each region was assigned to a single expert, who took the lead on the segmentation. However, the entire team met regularly to carefully review each segmentation, and each segmentation went through several iterations of refinements and reviews until the entire team was satisfied with the result. Neighboring regions were often assigned to different experts, further improving the robustness of boundary delineations.

Each atlas was segmented on a consistent contrast, using an auto-contrast feature in itk-SNAP, which removed the brightest and darkest voxels. It removed the first 0.5% of the voxels, on both extremes, to improve the overall contrast. The data available on NITRC will be adjustable according to the software the user is employing.

The P5 atlas had thirty-nine regions segmented; the P14, forty-five; the P72, twenty-nine. The differences in segmented structures between atlases were due to varying fields of view between subject ages, resulting in the P14 and P5 atlases having a slightly better contrast than the P72 atlas. The P5 and P14 brains were much smaller than the P72 brain, thus the scan time was spent over a smaller area, yielding better resolution and signal to noise ratio. As a result, several subcortical structures that were not visible in the P72 template were visible in the P5 and the P14 templates.

#### Segmentation of the P72 atlas

**Figure 1:**
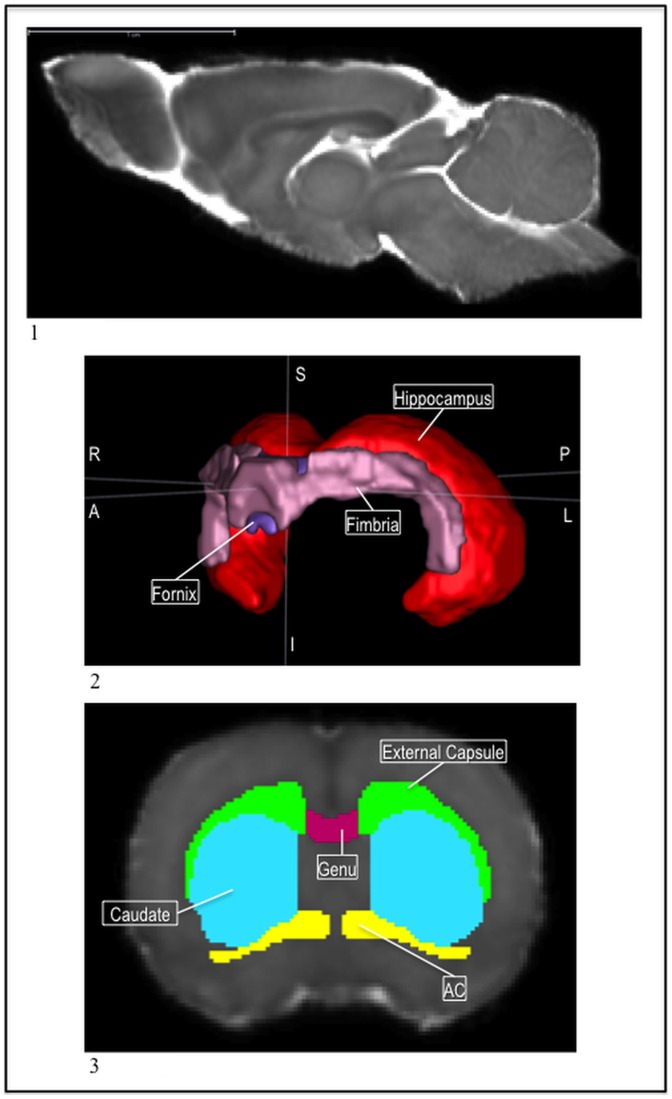
1) The P72 template MD image. 2) The P72 hippocampus (red), fimbria (pink), and fornix (purple) visualized as a 3D mesh. 3) The P72 caudate (blue), external capsule (green), genu (pink), and ac (yellow) in the MD.

**Figure 2:**
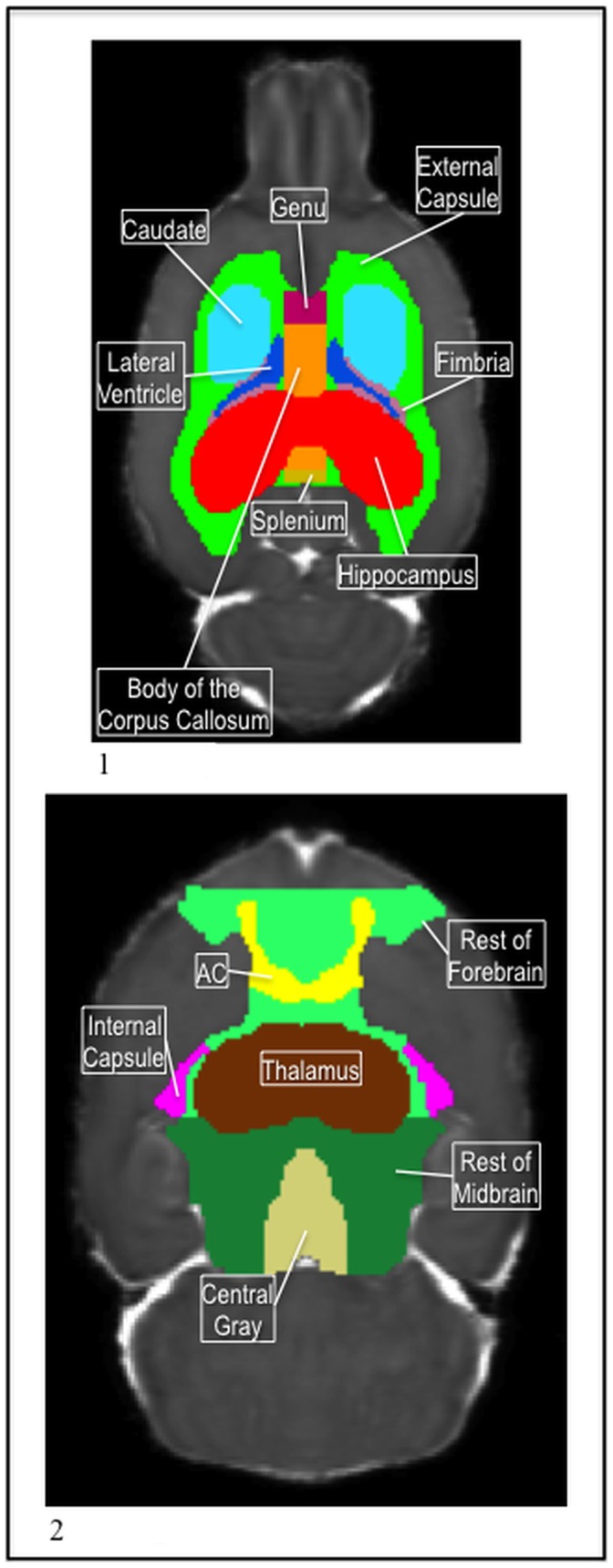
1) The P72 body of the corpus callosum (orange), genu (dark pink), and splenium (yellow), with the external capsule (green), caudate (light blue), lateral ventricles (dark blue), fimbria (light pink), and hippocampus (red) in the MD. 2) The P72 rest of forebrain (light green) and the rest of midbrain (dark green) with the ac (yellow), internal capsule (pink), thalamus (brown), and central gray (tan) in the MD.

Twenty-nine regions were segmented on the P72 MD template (Figure 1;1). The hippocampus was segmented early in the process since it had clear contrast and was easily distinguishable from the surrounding tissue. It was primarily segmented in the coronal view because that perspective provided a clear cross section of its tissue. After segmenting, the shape of the hippocampus was verified with the 3D meshing function in itk-SNAP (Figure 1;2).

**Figure 3:**
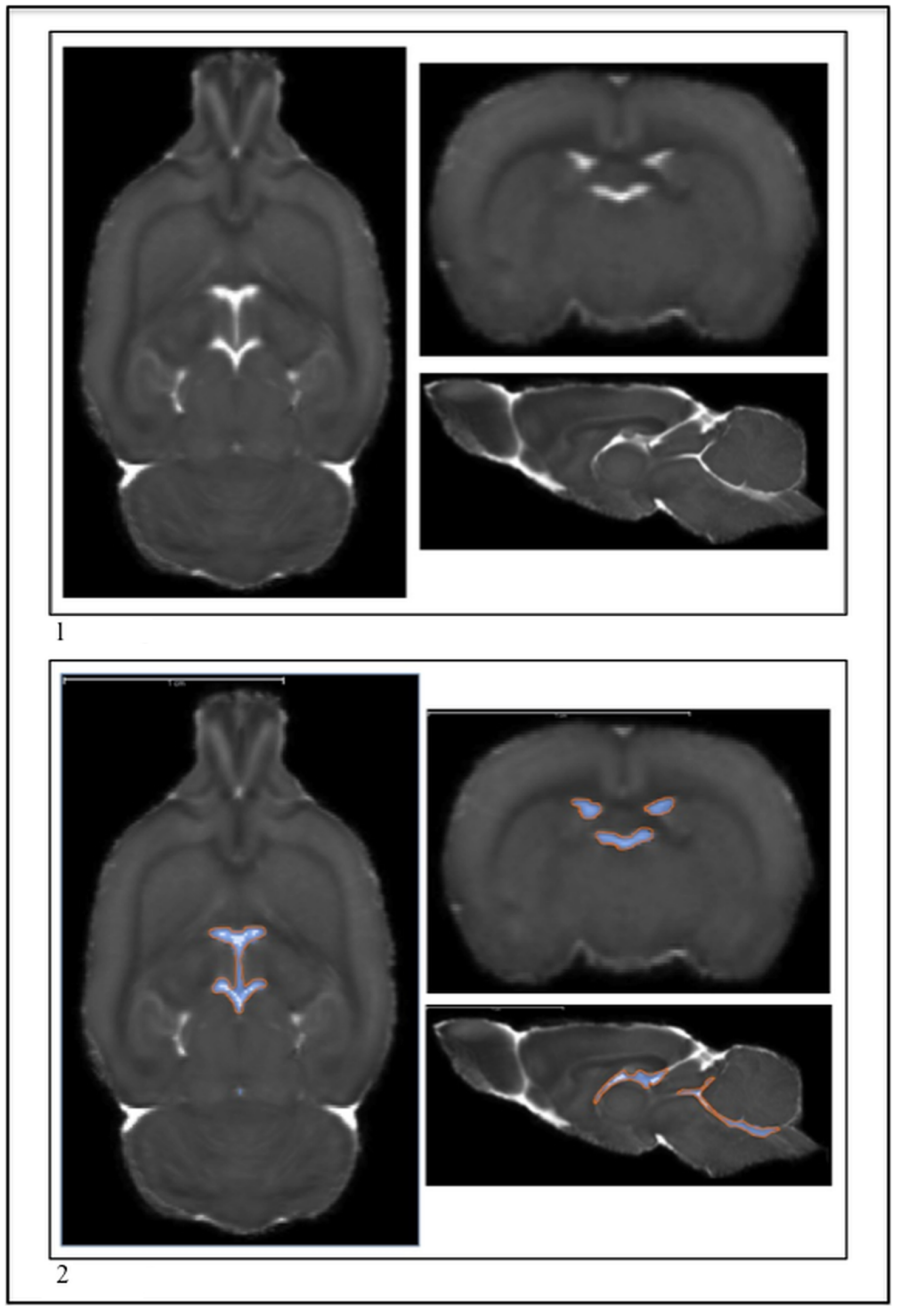
1) The P72 ventricle system before segmentation in the MD. 2) The P72 ventricle system after segmentation in the MD.

A white matter structure, the external capsule presented a very low intensity in MD images compared to the surrounding gray matter structures. Thus, the external capsule was one of the first regions segmented, in order to utilize it as a landmark for other structures, as it runs through much of the brain. The coronal view was used first to segment the external capsule running anterior to posterior; for the most part, it made a lowercase ‘m’ shape that was a lower MD intensity than the surrounding tissue, whether cortex or subcortical structure (Figure 1;3).

Originally, the entire external capsule and corpus callosum was segmented with one label, then later differentiated into the smaller regions in the corpus callosum as well as the left and right side of the external capsule. The genu of the corpus callosum stopped on the coronal slice before the lateral portions of the ac joined in the middle of the brain. The body of the corpus callosum transitioned to the splenium on the last coronal slice in which the medial portion of the hippocampus was connected. The genu, splenium, and the body of the corpus callosum did not extend past the lateral ventricles, thus on the most medial sagittal slice of the ventricles, the external capsule started (Figure 2;1).

Also a white matter structure, the ac presented lower intensity in the MD image than its surrounding tissue so it was easily distinguished and segmented. The anterior portion of the ac began once the forceps of the corpus callosum were visible in the tissue and extended posterior until a few slices before the lateral ventricles and the third ventricle connected in the coronal view. In the axial view, the ac had a horseshoe shape in the anterior portions as the anterior extensions gradually moved inward and medially, and connected across the midline. Then, as the structure continued posterior, it again developed projections, though these were not as long. It formed a landmark for the caudate label as well as helped us define the posterior boundary of the genu of the corpus callosum (Figure 2;2, Figure 1;3).

Like the external capsule and the hippocampus, the ventricles were easily distinguishable from the surrounding tissue because they presented a very high intensity in the MD image due to the high diffusivity in cerebral spinal fluid (CSF) (Figure 3;1, Figure 3;2). The lateral ventricles, the third ventricle, the aqueduct, and the fourth ventricle were labeled. Each served as an important landmark for nearby structures. For example, the aqueduct guided the segmentation of the central gray while the fourth ventricle made the boundary between the cerebellum and brainstem clear.

**Figure 4:**
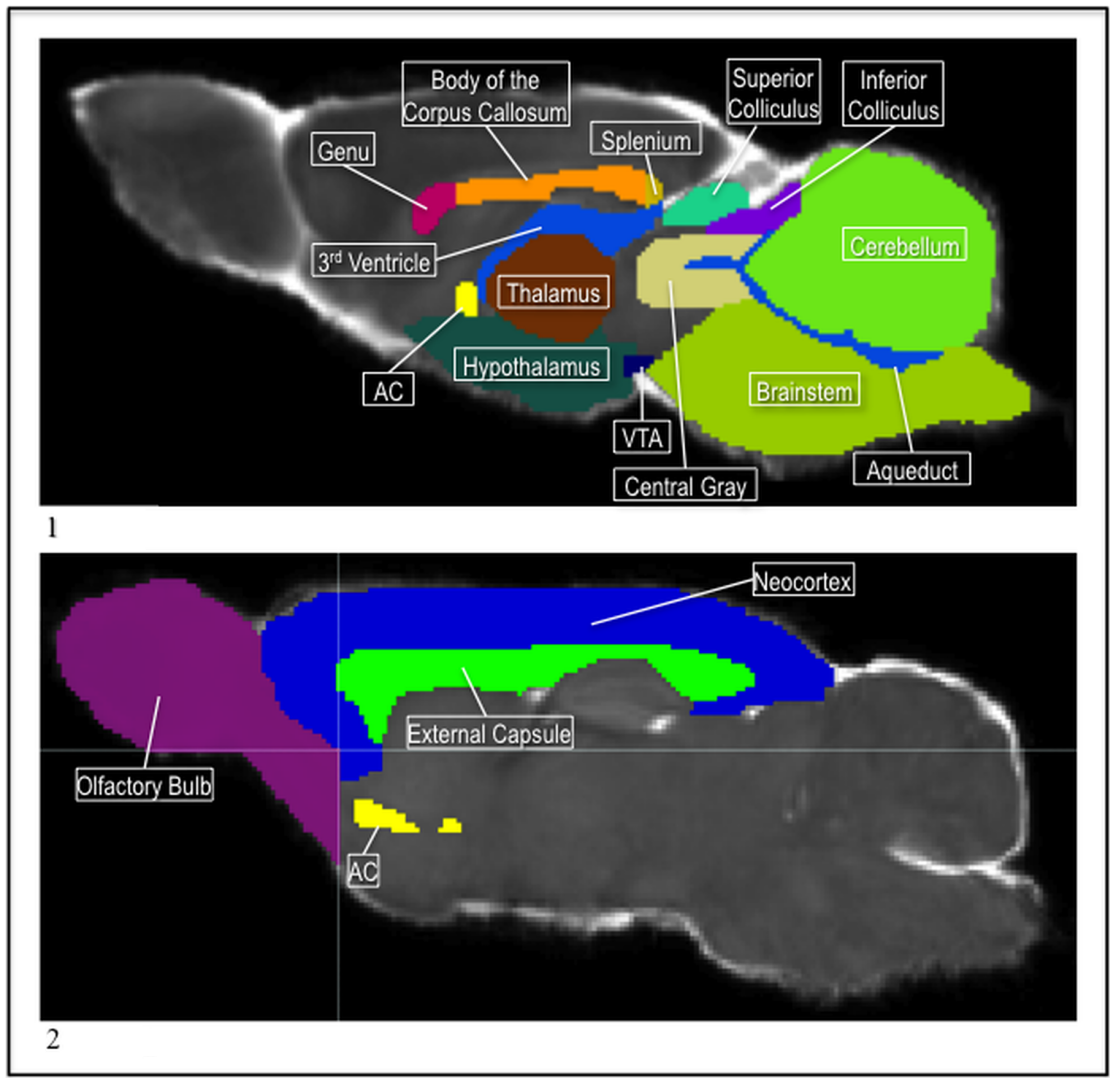
1) The P72 thalamus (brown), ac (yellow), hypothalamus (teal), VTA (navy), central gray (tan), superior (teal) and inferior (purple) colliculi, cerebellum (green), aqueduct, 3^rd^, and 4^th^ ventricle (blue), genu (pink), body of the corpus callosum (orange), splenium (gold), and brain stem (green) in the MD. 2) The P72 neocortex (blue), with the external capsule (green), ac (yellow), and olfactory bulb (pink) in the MD.

Next we segmented the cerebellum, the brainstem, and the olfactory bulb. We allowed the aqueduct and the fourth ventricle to define the anterior and inferior boundaries of the cerebellum (Figure 4;1). For the internal boundary of the brain stem, we drew a diagonal line in the sagittal view from the point of the cerebellum, where the aqueduct and fourth ventricle connect, to the edge of the brain where the hypothalamus ends and the pituitary gland sits. Then, we used the coronal view to make the boundary smooth between the brainstem and surrounding tissue. The olfactory bulb sits at the most anterior portion of the brain. In the coronal view, we used the anterior end of the ac and the emergence of the external capsule to help distinguish its posterior end from the neocortex label (Figure 4;2).

**Figure 5:**
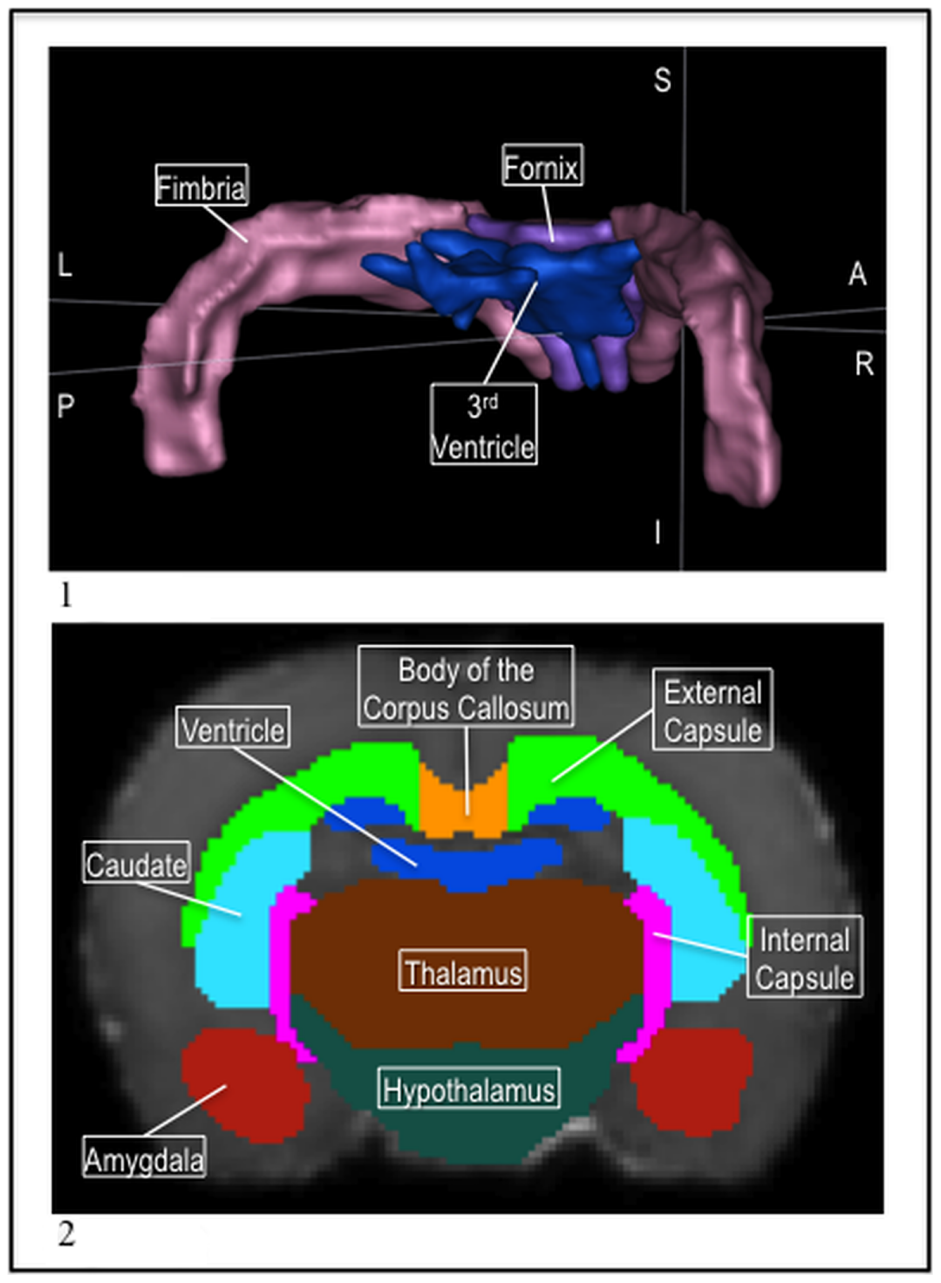
1) 2) The P72 fimbria (pink), fornix (purple), and 3^rd^ ventricle (blue) in a 3D mesh. 2) The P72 internal capsule (pink) and amygdala (red), with the external capsule (green), body of the corpus callosum (orange), 3^rd^ ventricle (blue), caudate (light blue), thalamus (brown), and hypothalamus (teal) in the MD.

Moving inward, the anterior subcortical structures that had strong enough contrast to be distinguished from the surrounding tissue or were near known landmarks in the brain, such as the fornix, fimbria, caudate, putamen, globus pallidus, internal capsule, and thalamus were segmented. Since the fornix and fimbria ran along structures that were easily distinguishable from the surrounding tissue, they were segmented first among these subcortical structures. For example, the fornix followed the shape and path of the third ventricle dorsally to the underside of the corpus callosum (Figure 5;1). Later, when the fimbria was added, it followed the path of the fornix up to the corpus callosum and the hippocampus. Once the hippocampus began, the fimbria split into lateral branches and ran posterior and inferior along each half of the hippocampus as it curved down (Figure 1;2).

The caudate, putamen, and globus pallidus were given a single label and kept cradled medially on each side of the external capsule by the ac and the internal capsule (Figure 1;3).The most anterior portion of the internal capsule started just posterior to the middle portion of the ac and ran inferior, staying medial to the caudate, through the forebrain and around the top part of the hypothalamus (Figure 5;2).

For the thalamus label, the sagittal view was primarily used for segmentation because it provided the best contrast of this structure against the surrounding tissue. The third ventricle helped distinguish its anterior and superior boundaries. The medial portion of the thalamus split when, in the coronal view, the internal capsule was no longer visible and the third ventricle began to extend downward before joining the aqueduct (Figure 4;1). The two lateral lobes continued posterior until the fimbria was no longer visible and the hippocampus was fully extended downward.

**Figure 6:**
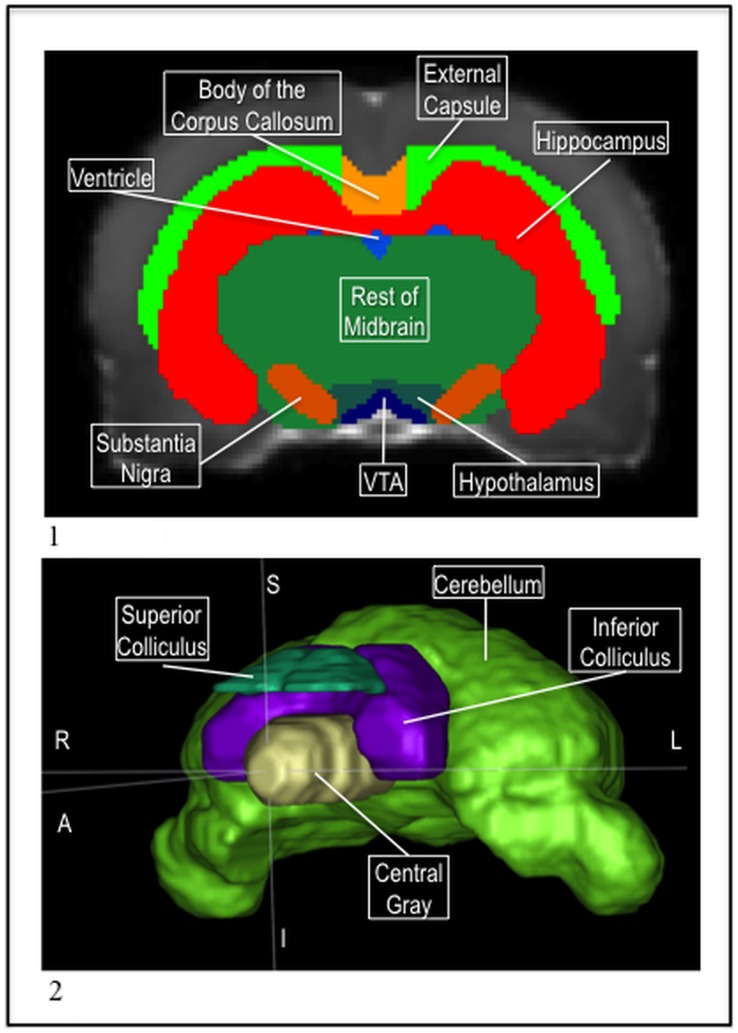
1) The P72 substantia nigra (orange) and VTA (navy) with the external capsule (light green), body of the corpus callosum (light orange), hippocampus (red), ventricles (blue), rest of midbrain (dark green), VTA (navy), and hypothalamus (teal) in the MD. 2) The P72 cerebellum (green), superior (teal) and inferior (purple) colliculi, and centrally gray (tan) in a 3D mesh.

Next, two subcortical structures in the ventral midbrain region, the ventral tegmental area (VTA) and the substantia nigra were segmented. The VTA sat medial to each substantia nigra, posterior to the hypothalamus region, and anterior to the brainstem. The VTA presented lower MD intensities than the hypothalamus and midbrain region, it appeared as an inverted “v” wedged between the substantia nigra (Figure 6;1). The substantia nigra started as the hypothalamus faded into the rest of midbrain label. They presented higher MD intensities than the surrounding tissue and their contrast was strong enough that they were able to serve as a landmark for the posterior end of the hypothalamus and the beginning of the VTA labels. They ran posterior from the hypothalamus along the lateral, ventral part of the midbrain and stopped before going into the brainstem.

After the lower midbrain structures, the superior and inferior colliculi and the central gray were marked. For the central gray, the aqueduct was followed from the cerebellum, anterior into the midbrain. The aqueduct helped define the shape of the central gray as it extended into the midbrain (Figure 4;1). The superior and inferior colliculi were first segmented in the sagittal view because the borders between these regions and the surrounding tissue were easier to distinguish and define. The colliculi remained superior to the central gray. The inferior colliculus sat just anterior to the cerebellum and the superior colliculus rested between it and the posterior ends of cortex. Also, the itk-SNAP 3D meshing function was utilized to visualize the structures in 3D to verify the correct shape and position in the brain of these regions (Figure 6;2).

The central gray, and superior and inferior colliculi, along with the amygdala and the hypothalamus, were the most difficult regions to segment because of the lack of contrast between the structures and their surrounding tissue. For the amygdala, lowering the opacity of the labels in itk-SNAP allowed more details of the tissue to be seen through the segmentation, and a histological atlas was consulted for all three views to verify its shape and position. The amygdala began at the posterior ends of the lateral projections of the anterior commissure, in the coronal view, and continued back until the fimbria connected the superior and inferior ends of the hippocampus and the lateral ventricles had ended. Also, the external capsule was used as a lateral boundary, making sure the amygdala did not go past it laterally, on each side in the coronal view (Figure 5;2). The hypothalamus rested on the bottom of the brain and ran from under the ac and the thalamus, to the substantia nigra and VTA (Figure 4;1, Figure 6;1).

Three of the labels, the rest of forebrain, rest of midbrain, and neocortex were created towards the end of the segmentation process so that all the voxels in the brain area would be assigned a label value. They covered regions that contained smaller structures and nuclei that did not have enough contrast to be segmented individually, but which where still needed as part of the whole segmentation. The internal capsule was used to distinguish between the forebrain and midbrain; the forebrain label switched to midbrain at the most posterior coronal slice of the internal capsule (Figure 2;2). The forebrain label ran from the posterior end of the internal capsule, anterior to the olfactory bulb. The midbrain label ran from the internal capsule, posterior to until the cerebellum and brainstem labels were touching. Finally, a neocortex label was created for the cortex regions and covered the rest of the area outside of the external capsule that was not labeled (Figure 4;2).

#### Segmentation of the P14 atlas

**Figure 7:**
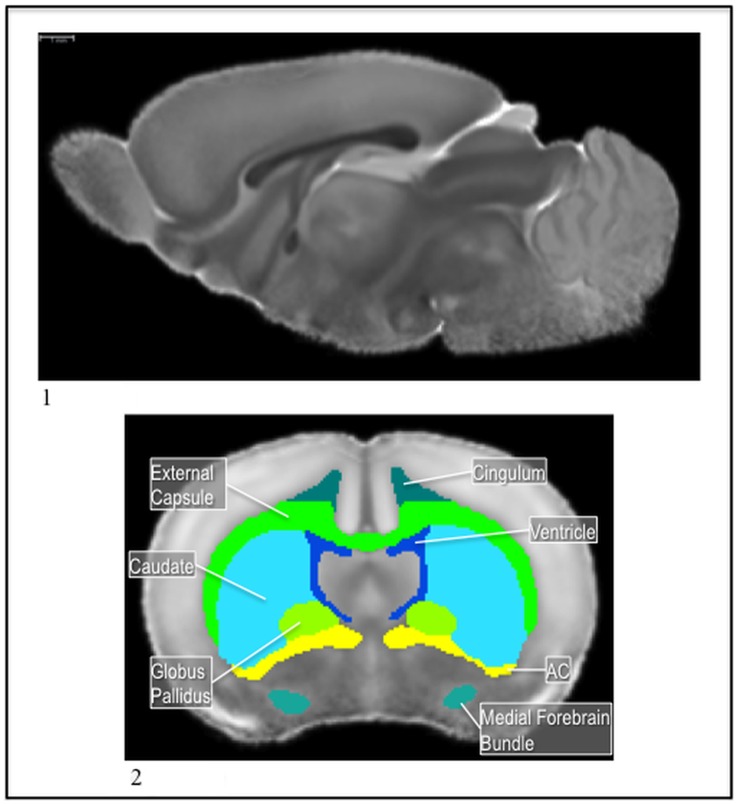
1) The P14 template MD image. 2) The P14 medial forebrain bundle (teal) and globus pallidus (lime green), with the caudate (light blue), lateral ventricles (dark blue), external capsule (light green), cingulum (dark green), and ac (yellow) in the MD.

The P14 atlas has forty-five segmented regions on the MD template image (Figure 7;1). The globus pallidus, the cingulum, the piriform, the red nucleus, and the medial forebrain bundle, which were not included in the P72 atlas, were added. Also, instead of a single neocortex label, the cortex was divided into sub-regions based on their anatomical location.

The globus pallidus lay on the medial underside of the caudate and followed the caudate posterior, from just before the ac joined across the midline until the hippocampus connected across the midline. It rested ventral to the lateral ventricles and dorsal to the lateral extensions of the ac (Figure 7;2). It presented a slightly lower MD intensity than the neighboring caudate tissue, while having a slightly higher intensity than the ac.

The cingulum sat on topmost part of the external capsule; in the coronal view, when the external capsule appeared as an “m,” the cingulum rested on the top of each arch (Figure 7;2). It was visible in the anterior portion as soon as the forceps of the corpus callosum extended downward and moved toward the midline to connect medially and ran posterior along the top of each side of the external capsule until the posterior end split again into the forceps of the corpus callosum. Another landmark for stopping the posterior extension of the cingulum was the appearance of the aqueduct in the central gray. The cingulum was distinguished visually because it had a slightly higher intensity than the external capsule, yet being white matter, had a lower intensity than the surrounding cortex regions.

**Figure 8:**
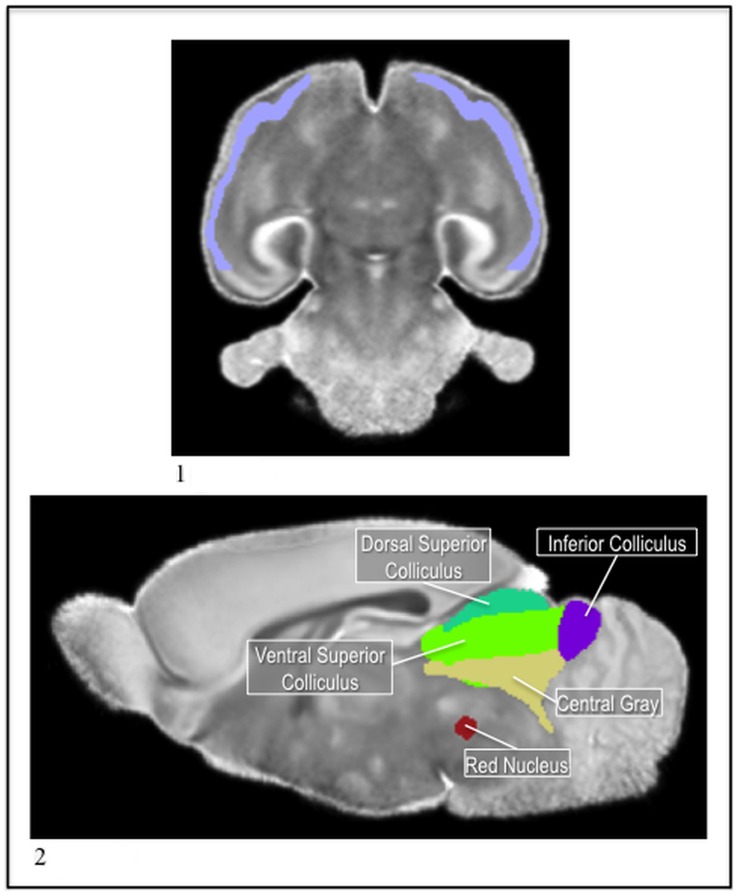
1) The P14 piriform in the MD. 2) The P14 red nucleus (red) with the superior (teal and green) and inferior (purple) colliculi, and central gray (tan) in the MD.

The piriform wound through the lateral ventral area of the cortex. It was easily distinguished from the surrounding cortex tissue because it presented a higher MD intensity and thus appeared as a brighter region in the cortex tissue (Figure 8;1). The piriform began in the coronal view one slice posterior to the olfactory bulb; the same slice where the external capsule was beginning to extend downward and medially and the ac’s lateral extensions were first visible. The piriform ran posterior along the most ventral and lateral part of the cortex. For its ending point, we followed the reference atlas and segmented the piriform such that the region was diminishing as the most anterior part of the aqueduct was emerging in the central gray, in the coronal view. Though the regions were not touching, we used the aqueduct as a landmark for the end of the piriform.

The red nucleus rested ventral to the central gray and colliculi; it began near the most anterior part of the aqueduct and ran posterior until the slice containing the most anterior bit of the inferior colliculus, in the coronal view. In both the sagittal and the coronal views it appeared as a small, circular region that presented a much higher MD intensity than the surrounding midbrain tissue (Figure 8;2). It was easily distinguishable because of its bright contrast and was able to serve as a landmark for the beginning of the inferior colliculus.

The medial forebrain bundle ran ventral to the caudate and thalamus, on the lateral sides of the hypothalamus. It had a higher MD intensity than the surrounding forebrain tissue and, as such, was easy to distinguish. Its segmentation began when the lateral ventricles began to extend downward before they joined across the midline in the coronal view (Figure 7;2). The medial forebrain bundle then ran posterior on each side of the hypothalamus, until the most anterior layers of the hippocampus were visible.

**Figure 9:**
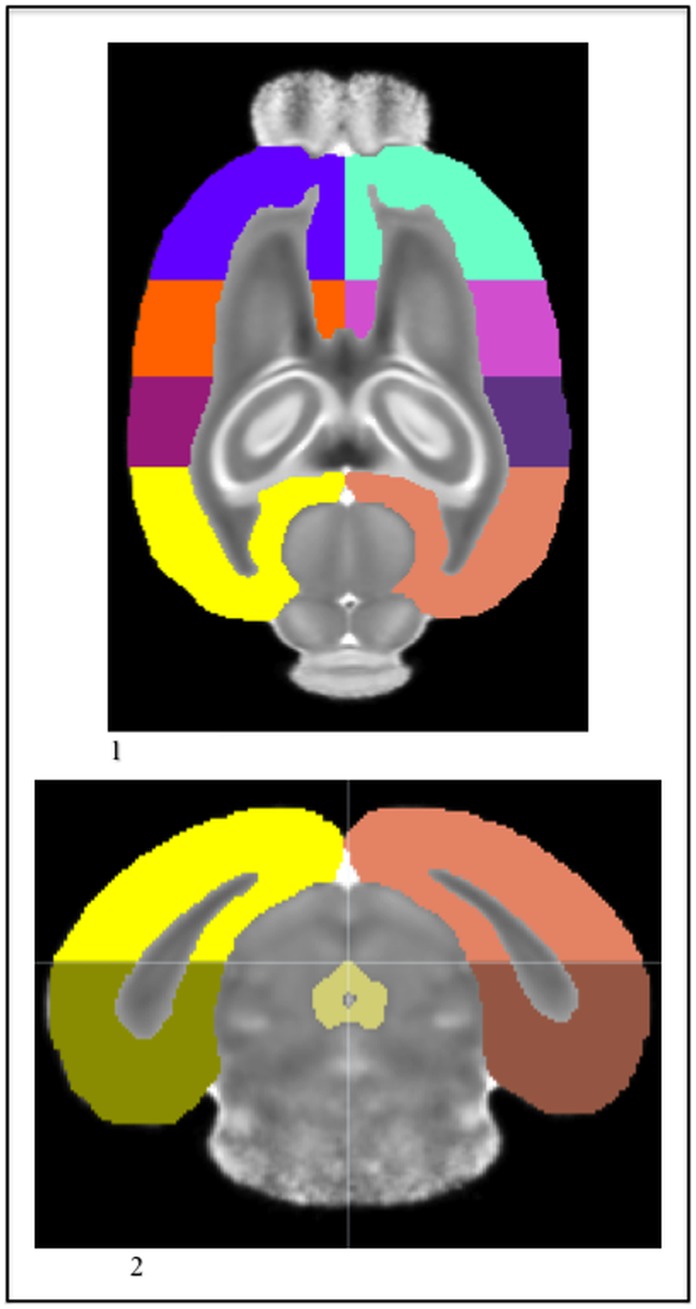
1) The P14 cortex divisions in the MD. 2) The P14 cortex divisions in the MD with the central gray (tan). The crosshairs allow the reader to see the dorsal (light) and ventral (dark) divisions as well as the lateral division.

The final addition to the P14 atlas was the subdivisions of the cortex. Instead of having one label for the entire cortex, anatomical directions were added to the labels to indicate the regions’ location. The divisions between these sub-regions were decided in the coronal view using landmarks to create the boundaries within the cortex (Figure 9;1, Figure 9;2). First, the left anterior cortex and the right anterior cortex were segmented from the emergence of the cortex above the olfactory bulb to the connection of the external capsule across the midline. The rest of the cortex regions had a dorsal and ventral label as well. The dorsal sections stayed above the central gray (Figure 9;2). In the axial view, when the central gray was first visible, the cortex labels switched from dorsal to ventral. The left anterior medial cortex and the right anterior medial cortex ran from the connection of the corpus callosum across the midline to the anterior point of the hippocampus. From there the left posterior medial cortex and the right posterior medial cortex ran to the last slice where the external capsule was connected across the midline. Then the left posterior cortex and the right posterior cortex began on the next slice and ran until the posterior end of the cortex above the colliculi.

We chose to distinguish between the dorsal and ventral regions of the cortex so that we could more accurately pinpoint statistical changes throughout the cortex in future data sets. Since these atlases are comprised of DTI data, it is possible that certain areas of the cortex may have greater or lesser diffusion than others, depending on the focus of the study. Having the cortex divided into smaller regions allows the researcher to more precisely locate changes in diffusion throughout the cortex as each region will have its own MD and FA values. Researchers not interested in such questions can simply combine these subdivisions to suit their own needs.

#### Segmentation of the P5 atlas

**Figure 10:**
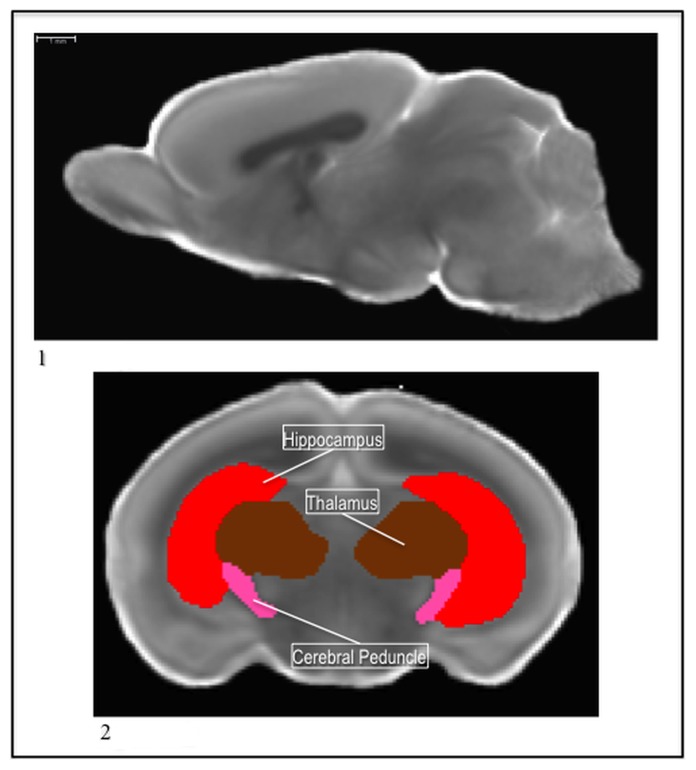
1) The P5 template MD image. 2) The P5 cerebral peduncle (pink) with the hippocampus (red) and the thalamus (brown) in the MD.

The P5 atlas had thirty-nine regions segmented on the MD template image (Figure 10;1). The cerebral peduncle, the pons, the columna fornicis, the nucleus accumbens, and the islands of Calleja were segmented uniquely to this atlas. Additionally, like in the P14 atlas the cortex was divided into anatomical sub-regions. Though the contrast in this template seems relatively poor next to the P14 atlas, in light of their improved resolution compared to the P72 atlas, it is important to keep in mind the young age of the animals and the underdevelopment of the structures. Most of the structures are still maturing at this early developmental point.

The cerebral peduncle was segmented from its emergence at the ventral extension of the internal capsule, as it ran posterior, to the most anterior part of the brain stem. The emergence of the ventral portion of the lateral ventricle, in the coronal view, was used as the landmark to begin segmenting the cerebral peduncle. As a white matter fiber bundle, it presented a lower MD intensity than surrounding midbrain tissue. When it first became visible, in the coronal view, it had a round shape, but as it ran posterior, it formed a diagonal slant on each side of the thalamus. In the anterior portion, each side formed a lateral cradle for the thalamus, and once the hippocampus extended downward, the cerebral peduncle remained wedged medially inside the lower sections of it (Figure 10;2).

**Figure 11:**
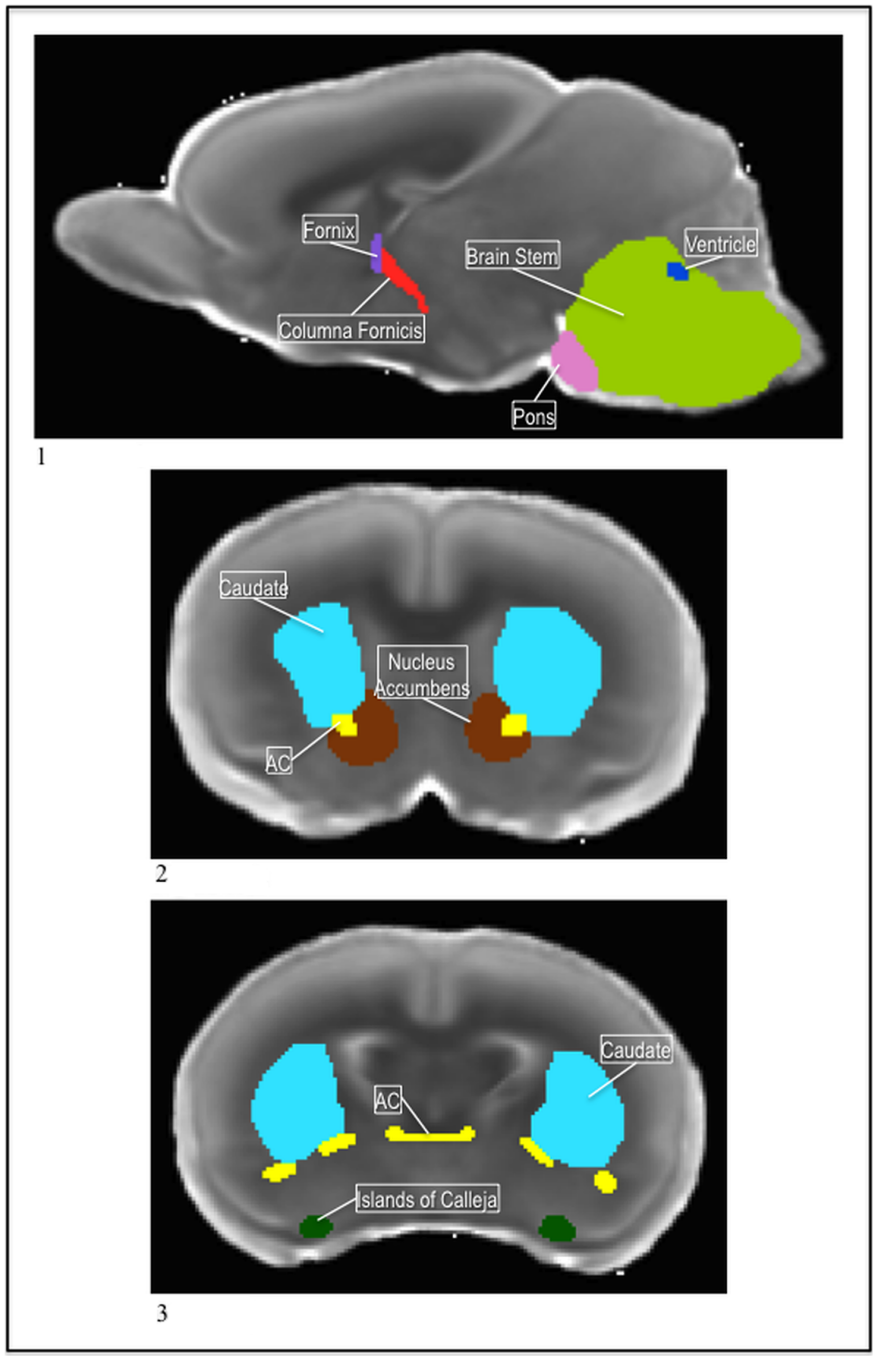
1) The P5 fornix (purple) and columna fornicis (red), with the pons (pink), brainstem (green), and ventricle (blue) in the MD. 2) The P5 nucleus accumbens (brown) with the caudate (blue) and the ac (yellow) in the MD. 3) The P5 islands of calleja (green) with the caudate (blue) and ac (yellow) in the MD.

The pons sits just anterior to the brainstem and posterior to the hypothalamus. As the brain stem emerged around it, in the coronal view, the pons presented a higher MD intensity, so it was easily distinguishable from the rest of the brainstem (Figure 11;1). As the pons ran posterior, the brain stem filled in around it and the pons ended around the midline in an upside down “v” shape.

The ventral projections of the fornix were labeled the columna fornicis. They were narrow extensions that ran laterally and ventrally from the body of the fornix through the hypothalamus until the emergence of the aqueduct. The coronal view was used to determine where to transition from the fornix label to the columna fornicis label. On the first slice where the fornix split laterally, on either side of the midline, we began the columna fornicis; the fornix label covered only the area where it was not separated laterally. The columna fornicis, like the fornix, presented a low MD intensity and thus was darker than the surrounding tissue, making it fairly easy to visualize (Figure 11;1).

The nucleus accumbens sat medial to the anterior projections of the ac. In the coronal view, each side had a “c” shape and wrapped around the extensions of the ac (Figure 11;2). In the sagittal and axial views, it had a round shape. The nucleus accumbens presented a higher MD intensity than the ac, so following the projections of the ac, it was easy to distinguish and label. The anterior portion of the nucleus accumbens emerged as the forceps of the corpus callosum extended downward, reaching for the midline. As the caudate emerged, the nucleus accumbens ran posterior until the extensions of the ac moved medially to connect across the midline.

The islands of Calleja sat on each lateral side of the brain near the ventral part of the cortex, and just medial to the edge of the piriform. In the coronal view it was a small, circular region that began just as the ac was branching into its posterior extensions and ended shortly posterior to those extensions, as the third ventricle connected across the midline (Figure 11;3). It presented a higher MD intensity than the immediately surrounding tissue, similar to that of the piriform.

## Discussion

MRI and DTI are non-invasive technologies that provide a wealth of 3D information about tissue that is not available via traditional imaging methods. However, the analysis of these datasets is relatively difficult if carried out manually. The field is at a stage where the acquisition of data happens at a faster rate than researchers can manually analyze. Thus, automated analysis methods are therefore crucial to make full use of these high-throughput imaging technologies. Additionally, the availability of age-appropriate MRI/DTI atlases is essential for automated analysis methods. Thus, we have presented three novel DTI atlases for rat brains at specific developmental time points, P5, P14 and P72. For each atlas, an unbiased average of the population was created and was used as a template for our anatomical experts to segment the individual brain structures. These atlases, containing an MD template, an FA template, a corresponding segmentation, a text label file, and a list of structure volumes, are made available to the research community via NITRC (http://www.nitrc.org/projects/dti_rat_atlas/).

There are dramatic differences between the three atlases, in addition to global scale differences due to the growing brain size: the white matter structures are still maturing in the P5 and P14 atlases; for example, the cerebellum and the hippocampus are considerably underdeveloped at P5, compared to P14 and adulthood. Because these atlases were created in a developmental succession, it is important to remain aware of the changes between each atlas and to select the appropriate atlas for use in one’s data analysis. Using an atlas that is not well matched to the data, may result in inaccurate analysis and distortions in the individual segmentations. Since the brain does not undergo dramatic change or development after adolescence, or P45 in rodents, the P72 atlas should remain appropriate for most adulthood data [24]. We have successfully used it on data sets ranging from P42 to P220. The P5 and P14 atlases will likely work on most data that is within 90% of the total brain volume of the rats that comprised each atlas. Using total brain volume instead of a strict postnatal day marker ensures the data will be closer in size to the atlas and therefore, more likely to be in a similar developmental state. Additionally, since volume gain reflects growth and development, using a brain volume window will allow the data to be similar in size and age to the atlas. It’s important to note that the age ranges suggested to be appropriate here may not be specific enough for many projects. Furthermore, regarding the use of the atlases on other rat strains, it is critical that the researcher make sure that atlas correctly fits his or her data in shape, size, and developmental stage. In order for the atlases to be applied successfully, the researcher must be able to achieve an accurate registration of the segmentation to the data, regardless of the age or strain. Thus, researchers must verify the accuracy of atlas-based registration and segmentation for each study to insure the atlas used is appropriate.

Because the pup brains are smaller than adult, we were able to achieve higher resolution in the same amount of scan time. This, combined with the different levels of tissue maturation (e.g. extent of myelination which affects FA contrast) which leads to different MR contrast has resulted in varying levels of detail being reliably resolved in the three age groups. For example, the P14 atlas includes the medial forebrain bundle, the red nucleus, the globus pallidus and the piriform, which we were not able to reliably segment in the adult atlas; similarly, the P5 atlas has the columna fornicis, the cerebral peduncle, the nucleus accumbens, the islands of Calleja, and the pons segmented. However, though the resolution was better in the P5 and P14 scans because the brains were smaller, the signal in the P5 and P14 scans was weaker because of their young age, and as such, we needed more images to achieve a stable template for those atlases compared to the P72 template.

We have already successfully used these atlases in our own studies (unpublished at the time of submission of this manuscript) for atlas-based segmentations in pup and adults in a study of the effects of gestational cocaine exposure (at ages P5, P14 and P72, in vivo scans of Sprague-Dawley rats) as well as an adolescent ethanol exposure study in adults (at ages P72, P80, P90 and P128, postmortem scans of Wistar rats). This was done by using a deformable registration method to compute a mapping between the subject scans and the atlas, and using the inverse map to carry the segmentation to the subject space [22]. The applicability of the atlas in different age groups, for mixed genders, strains, scan types, and both in control animals and in animals with a wide range of neurological defects further demonstrates the usefulness of the atlases. It should also be noted that the quality of the segmentation result would be heavily dependent on the registration quality for such an approach. Improved registration algorithms for mapping subject scans to the atlases we provide will lead to better segmentation results on the individual scans. This further illustrates that these atlases will be useful regardless of the particular segmentation method and will remain useful as new registration algorithms continue to be developed in the future.
